# Early echocardiographic signs of cardiovascular affection in pediatric familial hypercholesterolemia

**DOI:** 10.1007/s00431-023-05094-x

**Published:** 2023-07-21

**Authors:** Hossam Ibrahim, Hend Saad, Osama Abdelaziz, Gaser Abdelmohsen

**Affiliations:** Pediatric Cardiology Division, Department of Pediatrics, Faculty of Medicine, Specialized Pediatric Hospital, Kasr Al Ainy School of Medicine, Cairo University, Kasr Al Aini St, Cairo, 11562 Egypt

**Keywords:** Pediatric, Familial hypercholesterolemia, Aortic stiffness, Speckle-tracking echocardiography, Aortic insufficiency

## Abstract

Familial hypercholesterolemia (FH) is a rare autosomal dominant genetic disorder caused by defective low-density lipoprotein (LDL) receptors or abnormal apolipoprotein B. FH raises the risk of premature atherosclerotic disease and cardiovascular death in young adults. However, cardiovascular affection in children needs to be more adequately studied. Our study aimed to evaluate the effect of hypercholesterolemia on the cardiovascular system of pediatric patients with homozygous FH using conventional and advanced echocardiographic parameters such as tissue Doppler imaging (TDI) and 2-dimensional speckle-tracking echocardiography (2D-STE). This case-control study matched 25 healthy children with 21 patients with homozygous FH. Both groups had conventional echocardiography, TDI, and 2D-STE. Myocardial velocities of the left and right ventricles, left ventricular strain, and aortic stiffness parameters were measured. The FH group had greater systolic blood pressure, dilated coronary arteries, and hypertrophied left ventricle (LV) compared to the control (*P* = 0.0001, *P* = 0.001, *P* = 0.01, respectively). The mitral E/E′ ratio was higher in the patient group than in the control group (*P* = 0.007), indicating LV diastolic dysfunction in patients. At the same time, LV systolic function evaluated by 2D-STE was comparable to that in the control group. The abdominal aorta circumferential strain and ascending aorta M-mode-derived strain were significantly lower in patients compared to those in the control (*P* = 0.024, *P* = 0.0001, respectively), indicating increased aortic stiffness in the patients’ group; moreover, 85.7% of patients had mild aortic insufficiency.

*  Conclusion*: Mild aortic insufficiency, coronary artery dilatation, left ventricular (LV) diastolic dysfunction, and increased aortic stiffness are among early cardiovascular markers in pediatric patients with homozygous FH before impaired LV systolic function.

**Table:** 

**What is Known:** • *Familial hypercholesterolemia (FH) in adults is associated with accelerated atherosclerosis, aortic valvopathy, dilated coronary arteries, ischemic heart disease, and premature cardiovascular death.* • *The cardiovascular effects of FH in children require additional research.*	
**What is New:** • *Pediatric patients with familial hypercholesterolemia tend to have an early affection for left ventricular diastolic function before the affection for the systolic function.* • *The diastolic dysfunction associated with pediatric FH is correlated to the aortic stiffness and low-density lipoprotein levels.*

**What is Known:**

• *Familial hypercholesterolemia (FH) in adults is associated with accelerated atherosclerosis, aortic valvopathy, dilated coronary arteries, ischemic heart disease, and premature cardiovascular death.*

• *The cardiovascular effects of FH in children require additional research.*

**What is New:**

• *Pediatric patients with familial hypercholesterolemia tend to have an early affection for left ventricular diastolic function before the affection for the systolic function.*

• *The diastolic dysfunction associated with pediatric FH is correlated to the aortic stiffness and low-density lipoprotein levels.*

## Introduction

Familial hypercholesterolemia (FH) is an autosomal dominant genetic disorder associated with elevated levels of low-density lipoprotein cholesterol (LDL), which can cause atherosclerotic disease, cardiovascular consequences, and premature death. FH is classified into two categories, the homozygous and heterozygous variants. According to previous studies, the prevalence of homozygous and heterozygous variants in the general population is around 1 in 1,000,000 and 1 in 500,000, respectively. FH is caused by mutations in LDL receptors, apolipoprotein B, or proprotein convertase subtilisin/kexin type 9 [1, 2]. Hypercholesterolemia can result in endothelial dysfunction and morphological vascular abnormalities, such as an increase in the intima-media thickness of peripheral arteries. Young adults with FH can potentially develop myocardial ischemia and LV dysfunction due to coronary artery stenosis [3, 4]. Previous studies reported early subclinical global systolic and diastolic LV dysfunction in hypercholesterolemic patients without coronary artery disease using conventional echocardiography, tissue Doppler imaging (TDI) and 2D-speckle-tracking echocardiography(STE) [5, 6].

Some publications have discovered familial hypercholesterolemia (FH) is related to a remarkable decrease in arterial compliance compared to normocholesterolemic healthy individuals [7]. Cardiovascular complications of FH in the pediatric age group have yet to be thoroughly studied, and there have been few studies in the pediatric age group. Our study aims to evaluate the effect of hypercholesterolemia on the cardiovascular system in pediatric patients with *homozygous familial hypercholesterolemia*, including systolic and diastolic cardiac functions and aortic stiffness, using conventional and advanced echocardiographic techniques like TDI and 2D-STE.

## Methods

**Fig. 1:**
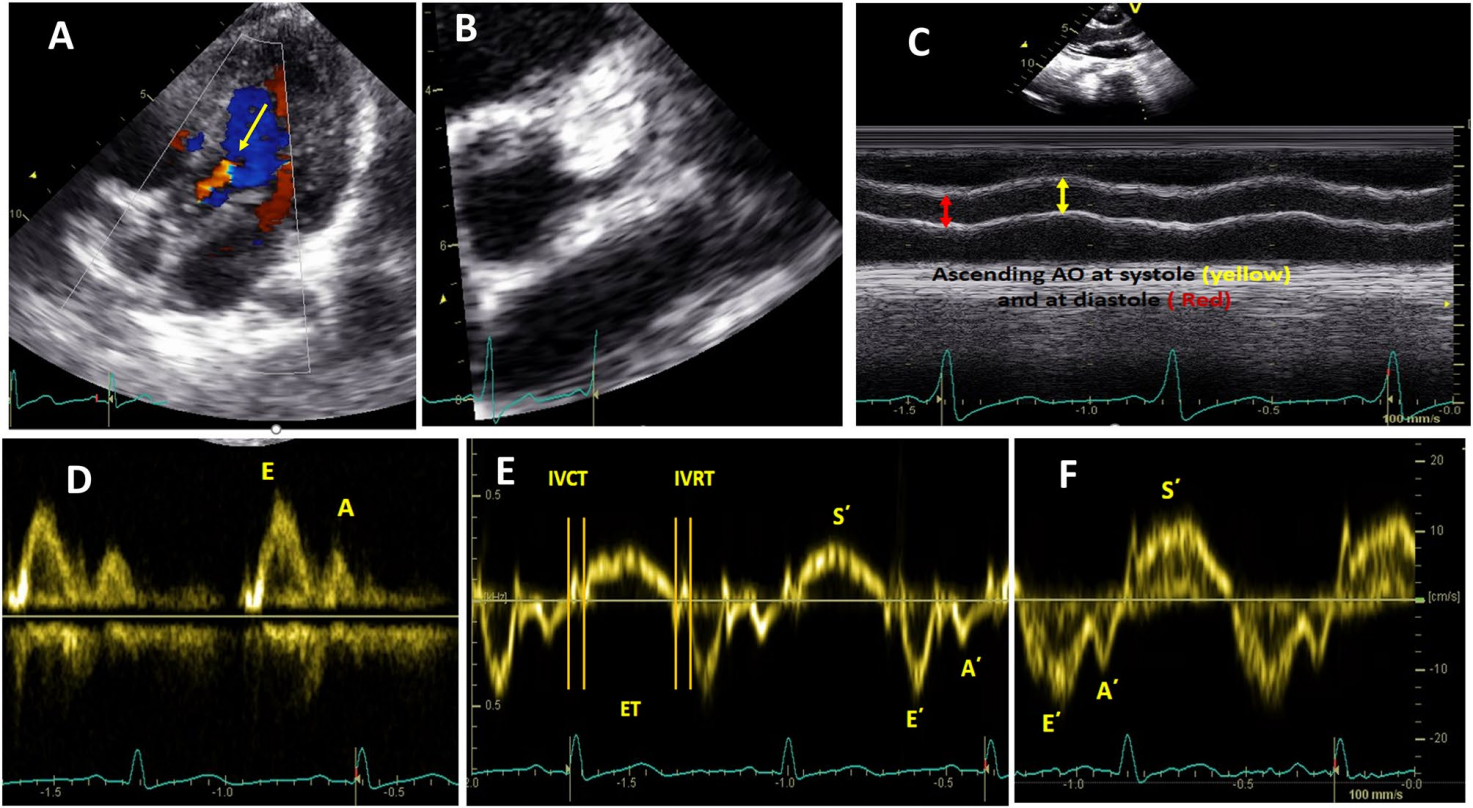
Conventional echocardiography and TDI done for the studied patients. **A** Apical five-chamber view with color Doppler showing mild aortic insufficiency (yellow arrow) in one patient with homozygous FH. **B** Parasternal short-axis view with zoom on left coronary artery showing left main coronary artery ectasia. **C** Calculation of ascending aorta strain using M-mode at ascending aorta in the parasternal long-axis view, then measuring systolic (yellow arrows) and diastolic (red arrows) aortic dimensions. **D** Pulsed Doppler at mitral inflow showing early (E) and late (A) mitral inflow velocities. **E** Pulsed wave TDI at the basal part of the LV lateral wall showing myocardial velocities and time intervals. **F** Pulsed wave TDI at the basal part of the RV lateral wall showing myocardial velocities. AO aorta, ET ejection time, FH familial hypercholesterolemia, IVCT isovolumic contraction time, IVRT isovolumic relaxation time, LV left ventricle, RV right ventricle, TDI tissue Doppler imaging

**Fig. 2:**
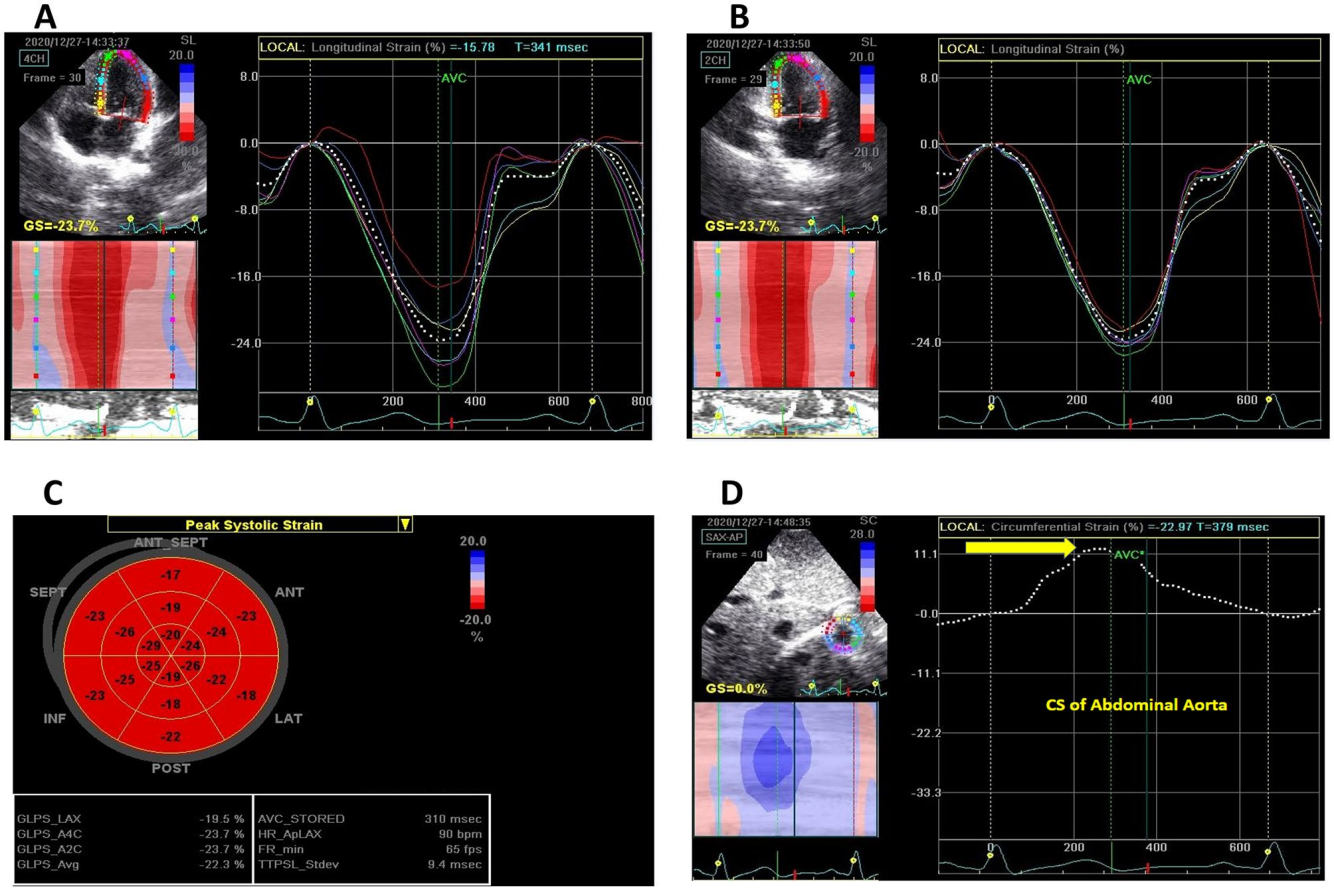
2D-STE done for patients' group. **A** LV longitudinal strain measured at the four-chamber view. **B** LV longitudinal strain measured at the two-chamber view. **C** Bull’s eye segmental and global LV longitudinal strain. **D** Abdominal aorta CS. CS circumferential strain, GS global strain, LV left ventricle, STE speckle-tracking echocardiography

Care providers’ informed consent was obtained, and the local ethical committee approved the study. This case-control study included 21 children with familial hypercholesterolemia and 25 healthy controls. Patients’ clinical and laboratory data were collected (Table 1), and echocardiography was performed for both groups. Expert pediatric cardiologist in modern echocardiographic techniques such as TDI and 2D-STE performed all cases in supine and left lateral positions utilizing a General Electric (GE, Vivid-7) system with probe 3 or 5 MHz (multifrequency transducer) based on patient age. Using the ECG cable, the beginnings of QRS complexes were used to characterize and time cardiac cycle events. Echocardiographic data collected were the following:

1. Conventional Doppler: Color Doppler was used to evaluate the degree of aortic insufficiency (Fig. 1A), and pulsed Doppler was used to measure blood flow velocities. The apical four-chamber view was used for the mitral and tricuspid valve inflow Doppler and the apical five chambers for the aortic valve Doppler (Fig. 1D).
2. M-mode: It is used for the measurement of LV dimension, left atrial dimensions, aortic systolic and diastolic dimensions (measured at the level ascending aorta after the sinotubular junction in parasternal long-axis view, Fig. 1C), and tricuspid annular plane systolic excursion (TAPSE).
3. TDI: To improve temporal resolution, sector width and depth were decreased to boost the frame rate to over 180 frames/s. During measurements, the ultrasound interrogation beam was aligned parallel (interrogation angle < 15°) to the target wall. Three cardiac cycles were recorded, and the average velocity and time were estimated to limit the effect of respiration on tissue velocities since breath-holding is not possible in small children. The measured parameters included systolic (S′) and diastolic (E′, A′) myocardial velocities, the isovolumic contraction time (IVCT), and isovolumic relaxation time (IVRT) and ejection time (ET) at the base of the LV lateral wall, septal wall, and the basal part of the right ventricle (RV) free wall (Fig. 1E–F). The myocardial performance index (MPI) was also measured for LV and RV using this formula: MPI = (ICT + IRT)/ET, as shown in Fig. 1E.
4. 2D echocardiography was used for the evaluation of coronary artery dimensions (Fig. 1B) and the evaluation of 2D-STE. Regarding STE, 2D images of the apical four chambers, apical long axis, and apical two chambers for LV longitudinal strain were taken (Fig. 2A–C). For LV, circumferential strain images were taken at parasternal short-axis view at the LV base, papillary muscles, and apex. Care was made to maintain posterior wall thickness throughout the cardiac cycle and avoid cuts into the left atrium due to translational heart movement. The frame rate was set from 60 to 90 Hz frame rates. The EchoPAC (EchoPAC version 11, GE) software is used for analysis. Cardiac cycles with lengths greater than 10% different from the mean length of the three cardiac cycles were eliminated from further analysis. Endocardial boundaries were manually tracked, and epicardial borders were adjusted. Tracking was acceptable if the EchoPAC software and examiner indicated good tracking throughout the cardiac cycle. Q analysis-2D strain assessed segmental and global strains. The 18 Bull’s eye segments were used to compute the segmental longitudinal strain of the LV. The aortic circumferential strain was measured using the short axis of the abdominal aorta, and the average circumferential strain was measured (Fig. 2D).
5. Aortic (AO) stiffness parameters measured by echocardiography included the following: *(A): M-mode derived *strain measured using M-mode at the ascending aorta at the parasternal long axis, and aortic diameters during systole (As) and diastole (Ad) were measured (Fig. 1C). The aortic strain was calculated using the following formula: Ascending AO M-mode strain [(As − Ad)/Ad]. Aortic distensibility was also measured using this formula (2xstrain/Δ*P*), where Δ*P* is the difference between systolic and diastolic blood pressure. Aortic elastance was calculated using the following formula: aortic elastance = (Δ*P*/Δ*V*) where Δ*V* equals aortic systolic, diastolic dimension differences. *(B):2D or B-mode* for abdominal aorta short-axis view for the measurement of systolic area (*S*), diastolic area (*D*), and area change %. Aortic area change was calculated using the following formula: aortic area change, % = (*S* − *D*)/*D* [8].

**Table 1:** Characteristics of studied groups

	Patients (*n* = 21)	Control (*n* = 25)	*P* value
Age, years	9 (5.5–11.5)	9 (6–11)	0.824
Male/female, *n* (%)	11/10 (52.4/47.6)	11/14 (44/56)	0.571
Weight, kg	24 (17–37)	25 (20.5–35.5)	0.589
Height, cm	129 (108.5–145)	127 (103–134)	0.421
Body surface area, m^2^	0.92 (0.71–1.23)	0.94 (0.74–1.15)	0.860
Systolic blood pressure, mmHg	118 (107–120)	100 (90–100)	0.0001*
Diastolic blood pressure, mmHg	66 (60–70)	60 (60–66)	0.082
Family history of similar conditions, *n* (%)	21 (100)	-	
Xanthoma, *n* (%)	21 (100)	-	
Xanthelasma, *n* (%)	1 (4.8)	-	
Exercise intolerance, *n* (%)	10 (47.6)	-	
Chest pain with excretion, *n* (%)	1 (4.8)	-	
Palpitation, *n* (%)	3 (14.3)	-	
Syncope, *n* (%)	1 (4.8)	-	
Laboratory		-	
Total cholesterol (mg/dl)	661 (483–762)		
LDL cholesterol (mg/dl)	574 (415–639)		
HDL cholesterol (mg/dl)	45 (27.5–51)		
Triglycerides (mg/dl)	128 (90–160)		

*LDL* low-density lipoprotein, *HDL* high-density lipoprotein

^*^Statistically significant

### Statistical analysis

The Statistical Package for Social Science (SPSS, Chicago, IL, USA, version 19.0) was used for statistical analysis. The data were checked for normality and then expressed as the median and interquartile range (25th–75th percentile). Numbers and percentages were used to summarize categorical data. Comparison between groups was made using the non-parametric Mann-Whitney *U* test for non-normally distributed numeric data, the Student *T* test for normally distributed numeric data, and the chi-square test for the categorical variables. The correlation between variables was determined using Pearson and Spearman correlation coefficients. *P* values less than 0.05 were considered statistically significant.

## Results

### Demographic and clinical characteristics of studied groups

Patients and control groups were matched regarding age, weight, height, gender, and body surface area. The patient group had higher systolic blood pressure than the control group. All patients had xanthomas and positive family history. One had chest pain with activity, and patients also showed high total cholesterol and LDL levels. Table 1 illustrates the clinical features of the studied groups.

### Conventional echocardiography

Few patients had aortic stenosis (14.3%), while most FH patients had mild aortic insufficiency (85.7%). Based on 2D images and M-mode in the echocardiography, the left ventricle was significantly hypertrophied, and the right and left main coronary arteries were more dilated in patients than in the control. Fractional shortening (FS) and ejection fraction (EF) values were notably lower, while the left atrium (LA) dimension was higher in the patient group. TAPSE was comparable between patients and control. Table 2 shows the conventional echocardiographic data of patients and control.

**Table 2:** Conventional echocardiography of studied groups

	Patients (*n* = 21)	Control (*n* = 25)	*P* value
2D echocardiography			
LCA, mm	4 (3.8–4)	3 (2–3)	0.001*
RCA, mm	3 (3–3)	2.1 (2–2.5)	0.001*
M-mode			
IVSDD, cm	0.6 (0.55–0.700)	0.50 (0.50–0.60)	0.010*
LVPWDD, cm	0.50 (0.50–0.65)	0.40 (0.40–0.50)	0.001*
LVEDD, cm	3.90 (3.80–4.20)	3.80 (3.65–4.20)	0.368
LVESD, cm	2.50 (2.40–2.75)	2.40 (2.10–2.65)	0.067
FS, %	34 (32–40)	39 (36–41)	0.037*
EF, %	64 (61–71)	70 (67–74)	0.016*
AO, cm	1.90 (1.80–2.30)	2.00 (1.90–2.35)	0.297
LA, cm	2.50 (2.30–2.95)	2.20 (2.05–2.55)	0.029*
TAPSE, mm	23 (19–23.75)	21 (18–23)	0.296
PW Doppler			
Mitral E, cm/s	90 (81.50–101.50)	90 (82–102)	0.938
Mitral A, cm/s	59 (48.5–74.5)	51 (46.5–55)	0.052
Mitral E/A	1.60 (1.45–1.92)	1.53 (1.80–1.97)	0.220
Aortic valve stenosis, *n* (%)			
No	18 (85.7)		
Mild	1 (4.8)		
Moderate	2 (9.5)		
Mild aortic insufficiency, *n* (%)	18 (85.7)	-	

*AO* aorta, *EF* ejection fraction, *FS* fraction of shortening, *IVSDD* interventricular septum diastolic dimension, *LA* left atrium, *LCA* left coronary artery, *LVEDD* left ventricle end-diastolic dimension, *LVESD* left ventricle end-systolic dimension, *LVPWDD* left ventricle posterior wall diastolic dimension, *PW* pulsed wave, *RCA* right coronary artery, *TAPSE* tricuspid annular plane systolic excursion

^*^Statistically significant

### Tissue doppler imaging (TDI)

The LV showed some diastolic dysfunction in patients compared to that in control, as the Septal and LV lateral E′ velocities were significantly lower in patients. Moreover, the mitral E/E′ was higher in the patient group than in the control group (Table 3).

**Table 3:** Tissue Doppler, cardiac strain, and vascular stiffness parameters of studied groups

	Patients, *N* = 21	Control, *N* = 25	*P* value
TDI parameters of LV			
Septal S′, cm/s	8 (7–8)	8 (7.5–9)	0.095
Septal E′, cm/s	12 (11–13)	14 (13–15)	0.003*
Septal A′, cm/s	7 (6–8)	7 (6–7.5)	0.260
Lateral S′, cm/s	8 (7.5–9)	8 (7–9.5)	0.650
Lateral E′, cm/s	14 (12–19.5)	17 (16–20)	0.045*
Lateral A′, cm/s	7 (6–9)	7 (6.5–8)	0.848
Mitral E/lateral E′	6.45 (5.19–6.84)	5.16 (4.47–5.97)	0.007*
TDI parameters of RV			
S′, cm/s	14 (13–15)	14 (12–15)	0.412
E′, cm/s	16 (14–19)	17 (15–19)	0.556
A′, cm/s	13 (10.5–15.50)	10 (9–11)	0.002*
Left ventricle strain, %			
GLS	− 19.3 (− 17.2 to − 22.3)	− 21.4 (− 19.5 to − 21.9)	0.087
GCS	− 16.9 (− 15.6 to − 19.7)	− 17.7 (− 16.3 to − 20)	0.635
GRS	19 (17–25)	15.3 (17.5–22.8)	0.178
Aortic stiffness parameters			
Abdominal aorta CS, %	11.6 (8.55–14.75)	14.70 (12.1–16.8)	0.024*
Ascending AO M-mode strain [(As − Ad)/Ad]	14.29 (10.26–17.91)	26.67 (14.84–32.29)	0.0001*
Aortic distensibility (2xstrain/Δ*P*), (cm^2^ dyn^−1^ 10^−3^)	0.47 (0.36–0.73)	1.53 (1.04–1.95)	0.0001*
Aortic elastance (Δ*P*/Δ*V*)	166 (86–194)	100 (63–112)	0.003*
Aortic area change, %= (*S* − *D*)/*D*	40 (25–58)	62 (46–71)	0.012*

*AO* aorta, *GCS* global circumferential strain, *GLS* global longitudinal strain, *GRS* global radial strain, *LV* left ventricle, *RV* right ventricle, *TDI* tissue Doppler imaging, *CS* circumferential strain

^*^Statistically significant

### Speckle-tracking echocardiography (STE)

2D-STE measured the global and segmental longitudinal, circumferential, and radial left ventricular strains. There was no statistically significant difference between the patients and the control group regarding global or segmental LV strain parameters, indicating that global and segmental LV systolic function was preserved in both groups (Table 3).

### Aortic stiffness evaluated by echocardiography

Measuring the aortic stiffness of study participants revealed substantial differences between the sick and control groups. The FH group showed lower abdominal aorta circumferential strain (*P* = 0.024), ascending aorta M-mode strain (*P* = 0.0001), abdominal aorta area change (*P* = 0.0001), and aortic distensibility index (*P* = 0.0001). In contrast, aortic elastance was more significant in the patients compared to that in the control group (*P* = 0.003), indicating increased aortic stiffness in patients with FH as illustrated in Table 3.

Mitral E/E′ was correlated positively with the LDL cholesterol level and aortic elastance but negatively with circumferential aortic strain indicating that elevated LDL levels with the associated aortic stiffness are correlated to the subtle LV diastolic dysfunction, as shown in Table 4.

**Table 4:** Correlation between echocardiographic and laboratory variables

Correlated parameters	Correlation coefficient (*r*)	*P* value
Mitral E/E′		
LDL cholesterol	0.508	0.022*
Circumferential aortic strain	− 0.425	0.004*
Aortic elastance	0.387	0.014*

*LDL* low-density lipoprotein

^*^Statistically significant

## Discussion

Despite the prevalence of FH in children, few researchers have focused on the cardiovascular problems this condition might cause in young patients. This study aims to discover how hypercholesterolemia impacts the cardiovascular system in young children with homozygous FH.

### Clinical presentation in pediatric patients with FH

Hypercholesteremia increases vascular stiffness and elevates blood pressure by increasing the intimal endothelial thickness. This could explain the increase in systolic blood pressure measured in the patient group [3]. Xanthoma is one of the classical findings seen in cases with FH. In this report, clinical examination revealed the existence of xanthomas in all patients with FH, supporting the findings of Zak et al. who found a substantial correlation between xanthoma and the diagnosis of various kinds of dyslipidemia [9].

### FH and cardiac valvulopathy

FH can affect cardiac valves, especially the aortic valve, causing aortic valvopathy; in this cohort, 85.7% of patients had aortic valve insufficiency, while 14% had aortic valve stenosis. Valvopathy in FH usually affects the left-sided valves, especially the aortic valve. Kolansky et al. reported aortic regurgitation as the most common and first sign of FH valvulopathy and it was associated with angiographic coronary stenosis [10]; Kawaguchi et al. found that 8 of 10 homozygous FH patients had aortic valve stenosis that improved after lipid-lowering treatment and LDL apheresis [11]. The lower incidence of aortic stenosis in our study may be due to the young age of our patients. The first histological finding in aortic valvulopathy is cusp thickening due to lipid deposition, followed by a regurgitant jet from the center of coaptation without aortic annulus dilation. Inflammatory cells and lipid buildup thicken and disrupt the three cusps of the aortic valve [11].

FH can also cause supravalvular aortic stenosis. In untreated severe FH youngsters, Rafeiyian et al. found severe supra valvular aortic stenosis on echocardiography [12]. They also found a positive link between transaortic pressure gradients and integrated cholesterol exposure index (cholesterol level × year). The mechanical stress in the ascending aorta of individuals with increasing aortic stenosis and turbulent blood flow causes significant calcification in the aortic valve and the aorta [13].

### LV size and function in pediatric patients with FH

There was no statistically significant difference between patients and controls regarding LV systolic function parameters determined by TDI (S′ wave velocity), segmental or global strain values recorded by 2D-STE in this cohort. Unlike TDI and 2D-STE, the M-mode LV systolic parameters, FS and EF were significantly lower in patients compared to those in the control, with median values in the patient group of 34% (minimum 30, maximum 44) and 64% (minimum 60, maximum 75), respectively, which are still within normal limits for the pediatric age group. Mertens et al. reported that normal FS measurement varies between 28 and 38%, with values below 28% suggesting reduced systolic function and above 38% indicating hyperdynamic function [14].

Although considerable affection of longitudinal and circumferential deformation of the left ventricle in patients with FH was reported previously, this was not the case in our study. This discrepancy may be attributable to our cohort’s small sample size and younger mean age [15].

Patients in this group displayed LV hypertrophy compared to controls, probably because of higher afterload caused by arterial stiffness. Our patients’ greater systolic blood pressure could explain the reported LV hypertrophy.

Like previous studies, this cohort showed an early LV diastolic dysfunction in FH patients, as the mitral E/ E′ was higher in the patient group. Also, E/E′ was correlated to elevated levels of LDL [5, 15]. According to previous experimental research, two processes have been implicated in heart dysfunction in FH. First, an excess of cardiomyocyte lipids can have harmful consequences and cause cardiac dysfunction. In this concept, increased lipids in cardiomyocytes and lipid metabolites (e.g., ceramides, free fatty acids, lipid peroxides, acylcarnitine, diacylglycerols, long-chain acyl-CoAs, and lysophospholipids) induce toxicity via mitochondrial damage, ATP depletion, and sarcoplasmic reticulum stress [16]. An experiment showed that hypercholesterolemia decreases the quantity of connexin-43 protein, the primary gap junction component, and decreases cardiac conduction velocity, thus degrading ventricular contractile function [17]. This condition is referred to as “lipotoxic cardiomyopathy” or “cholesterol cardiomyopathy” [18]. Second, hypercholesterolemia increases oxidative/nitrative stress in the heart microcirculation by expanding the generation of reactive oxygen species and decreasing endothelial nitric oxide, resulting in endothelial dysfunction biosynthesis [19]. In addition, because these alterations occur at the microvascular level, they influence the contractile properties of the myocardium directly and independently of the development of atherosclerosis in major arteries. In the current study, diastolic dysfunction may account for the substantial enlargement of LA dimensions in patients compared to the control group.

### Coronary artery ectasia in FH

Coronary artery ectasia in our patients with FH was evident. Several reports found frequent coronary artery ectasia in patients with hypercholesterolemia [20, 21].

Thompson et al. showed improvement in coronary ectasia following plasma exchange in a patient with familial hypercholesterolemia, supporting a relationship between plasma lipoproteins and ectasia [22].

It has been established that the interaction of LDL with collagen and elastin promotes endocytosis by macrophages with the development of foam cells. After absorbing modified LDL, macrophages release elastase and collagenase, damaging coronary artery collagen and elastin fibers and causing ectasia. Immunohistochemical detection of apolipoprotein (apo) B, the major protein of LDL, close to collagen and elastin fibers in the intima, supports that hypothesis [23–25].

### Arterial stiffness in FH

In our study, there was a statistically significant difference between cases and controls in aortic distensibility and aortic change area, which were lower in the FH group than in the control group. This can be explained by the loss of elasticity of arterial wall due to the binding of LDL cholesterol particles to elastic fibers, the release of elastase enzyme by macrophages in atheromatous plaque areas, and the formation of free radicals due to oxidative stress [23, 24]

Aortic root and ascending aorta atheromatous plaques have been reported in FH. Furthermore, it has been revealed that the internal diameter of the supra valvular aortic ridge is smaller in FH patients than in healthy controls, resulting in decreased distensibility [11].

We examined the elasticity of the ascending aorta and the strain of the abdominal aorta in our study. Both were considerably impaired in patients and negatively linked with the mitral E/E′ ratio, indicating the possible effect of aortic stiffness on LV diastolic performance.

Some researchers have adopted STE to measure arterial strain as an index of arterial stiffness [26]. Cho et al. found that Peak CS of the aorta showed a good correlation with intima-media thickness and the reduced CS strain in the aorta with hypercholesterolemia. Still, they used CS of descending aorta by transoesophageal echocardiography, an invasive procedure [8].

### Limitations

One of the study’s significant limitations was the small number of patients recruited. Stress ECG, stress echocardiography, multidetector computed tomography, and coronary angiography were not performed on the patients, even though these modalities are critical for evaluating coronary arterial pathology. The assumed diastolic dysfunction was based on echocardiography and was not confirmed by interventional cardiac catheterization, the gold standard for assessing diastolic cardiac dysfunction. In contrast, cardiac catheterization for coronary angiography and hemodynamic evaluation is invasive.

## Conclusion

Aortic valve insufficiency, aortic valve stenosis, coronary artery dilatation, left ventricular (LV) diastolic dysfunction, LV hypertrophy, and increased aortic stiffness are among the early cardiovascular markers in pediatric patients with homozygous FH. These markers may appear before the deterioration of LV systolic function.

## Data Availability

The corresponding author can provide the data supporting this study upon reasonable request, providing patient data privacy is not violated.

## Abbreviations

AO: Aorta
CS: Circumferential strain
EF: Ejection fraction
ET: Ejection time
FH: Familial hypercholesterolemia
FS: Fractional shortening
GCS: Global circumferential strain
GLS: Global longitudinal strain
GRS: Global radial strain
HDL: High-density lipoprotein
IVCT: Isovolumic contraction time
IVRT: Isovolumic relaxation time
LA: Left atrium
LCA: Left coronary artery
LDL: Low-density lipoprotein
LV: Left ventricle
MPI: Myocardial performance index
RCA: Right coronary artery
STE: Speckle-tracking echocardiography
TAPSE: Tricuspid annular plane systolic excursion
TDI: Tissue Doppler imaging
